# Influenza Vaccination and Myo-Pericarditis in Patients Receiving Immune Checkpoint Inhibitors: Investigating the Likelihood of Interaction through the Vaccine Adverse Event Reporting System and VigiBase

**DOI:** 10.3390/vaccines9010019

**Published:** 2021-01-04

**Authors:** Milo Gatti, Emanuel Raschi, Ugo Moretti, Andrea Ardizzoni, Elisabetta Poluzzi, Igor Diemberger

**Affiliations:** 1 Department of Medical and Surgical Sciences, Alma Mater Studiorum—University of Bologna, 40138 Bologna, Italy; milo.gatti2@unibo.it (M.G.); emanuel.raschi@unibo.it (E.R.); elisabetta.poluzzi@unibo.it (E.P.); 2 Department of Public Health and Community Medicine, University of Verona, 37134 Verona, Italy; ugo.moretti@univr.it; 3 Medical Oncology Unit, Department of Experimental, Diagnostic and Specialty Medicine, Policlinico S. Orsola-Malpighi, Alma Mater Studiorum—University of Bologna, 40138 Bologna, Italy; andrea.ardizzoni2@unibo.it; 4 Cardiology Unit, Department of Experimental, Diagnostic and Specialty Medicine, Alma Mater Studiorum—University of Bologna, 40138 Bologna, Italy

**Keywords:** influenza vaccine, immune checkpoint inhibitors, myocarditis, pericarditis, pharmacovigilance, drug–vaccine interaction, adverse event following immunization

## Abstract

Background: Evidence on whether the influenza vaccine could exacerbate immune-related adverse events, including myopericarditis (MP), in patients treated with immune checkpoint inhibitors (ICIs), is still conflicting. We explored this issue through a global real-world approach. Methods: We queried the Vaccine Adverse Event Reporting System (VAERS) and VigiBase to retrieve cases of MP in which the influenza vaccine and ICIs were recorded as suspect and were concomitantly reported. For the included cases, causality assessment and Drug Interaction Probability Scale (DIPS) algorithms were applied. Results: There were 191 and 399 reports of MP with the influenza vaccine that were retrieved (VAERS and VigiBase, respectively). No case of MP reporting the concomitant use of ICIs and the influenza vaccine was found in VAERS, while three cases of myocarditis were retrieved in VigiBase. All of the cases were unclassifiable for a causality assessment because of the lack of data concerning latency. According to the DIPS, one report was categorized as possible and two as doubtful. Conclusion: The paucity of cases coupled with the doubtful causality assessment make the potential interaction between influenza vaccines and ICIs in cancer patients negligible from clinical and epidemiological standpoints. These findings support the cardiovascular safety of the influenza vaccination, which remains strongly recommended in cancer patients, especially in the current COVID-19 era.

## 1. Introduction

The safety of vaccines and medications is a current global safety issue. The annual vaccination against the influenza virus is the primary means of preventing influenza and its complications in high-risk subjects, including adult cancer patients, as described by observational studies, suggesting lower mortality and infection-related outcomes with influenza vaccination [1]. However, in the recent past, potential pharmacokinetic interactions have been proposed, considering that vaccines can influence the drug metabolism via inflammatory cytokines [2]. Therefore, potential interactions between influenza vaccines and drugs used for chronic diseases (e.g., immunosuppressive agents) cannot be excluded [3]. Moreover, cases of myo-pericarditis (MP) after immunization, although rarely, are reported [4,5].

Myocarditis and pericarditis represent serious and life-threatening inflammatory diseases involving myocardium and pericardium, potentially associated with the use of several drugs and vaccines [6,7,8]. In particular, cases of MP were described with the smallpox vaccine in early 2000 and, more recently, very rarely are reported after influenza immunization [4,5]. Immune checkpoint inhibitors (ICIs), such as anti-PD1, anti-PDL1, and anti-CTLA4 monoclonal antibodies, are approved as first-line agents in the management of melanoma and non-small cell lung cancer. They may cause a variegate spectrum of cardiovascular events, including MP, with a higher mortality compared with other immune-related adverse events (irAEs) [9,10,11,12,13]. In a summary of the product characteristics of the different ICIs, pericarditis and myocarditis are reported as uncommon (≥1/1000 to <1/100) and rare (≥1/10,000 to <1/1000, respectively) AEs. Notably, the detection of myocarditis with ICIs requires the permanent discontinuation of treatment, no matter the severity.

Consequently, the question arises as to whether pharmacokinetic and pharmacodynamic interactions occur in patients receiving the influenza vaccination (recommended both by oncologists and cardiologists) and ICIs, possibly causing or exacerbating irAEs such MP. Although no cases of myocarditis have been reported, evidence on whether the influenza vaccine exacerbates irAEs is still conflicting and poorly investigated [14]. Only a retrospective study investigated the clinical features of myocarditis with ICIs in patients receiving the influenza vaccine; reduced myocardial injury and a lower risk of major adverse cardiac events among recipients of the influenza vaccine was found compared with not vaccinated patients [15].

In the recent past, analysis of the spontaneous reporting systems (SRSs) has attracted considerable interest among clinicians for the accurate and timely characterization of drug- and vaccine-related risks occurring in the real world, where comorbidities and polypharmacotherapy exist. By offering a global epidemiological perspective, these pharmacovigilance studies have been pursued to test the hypothesis of potential associations, including refusing the likelihood of interactions [3,16,17], especially for rare, unexpected, and delayed AEs, such as MP.

In this study, we investigated the likelihood of interaction between the influenza vaccination and ICIs by analyzing spontaneous reports of MP collected from the Vaccine Adverse Event Reporting System (VAERS; P.O. Box 1100; Rockville, MD 20849-1100) and the World Health Organization’s (WHO) global Individual Case Safety Report database (VigiBase^®^; Uppsala Monitoring Centre Box, 1051 SE-751 40, Uppsala, Sweden).

## 2. Materials and Methods

### 2.1. Study Conception and Design

The study was conceived as an observational retrospective analysis of spontaneous reports of MP collected from the VAERS and the VigiBase^®^ to (a) characterize relevant clinical features; (b) highlight the concomitant use of agents known to cause MP, including ICIs; and (c) assess the causality and probability of interaction between the influenza vaccine and ICIs.

VAERS is a national system to monitor the safety of US-licensed vaccines [18], whereas VigiBase^®^ collects worldwide reports on vaccines and drugs, thus making these archives act as complementary approaches [19].

VAERS transmits its vaccine adverse event reports to the VigiBase, in order to contribute to the global pharmacovigilance effort along with other countries that employ passive vaccine safety monitoring systems [18], thus possible duplicates between the two databases may exist.

### 2.2. Data Source

Established in 1990, the VAERS is co-managed by the Centers for Disease Control and Prevention and the US Food and Drug Administration (FDA); it collects and analyzes reports of AEs following immunization (AEFI) for vaccines licensed in the US, receiving approximately 28,000 reports of AEFI annually.

AEFI may be any unfavorable or unintended sign, abnormal laboratory finding, symptom, or disease that occurs following or during the administration of a vaccine. AEs are temporally associated events that may either be caused by a vaccine or be coincidental to it, i.e., not necessarily related to the vaccination [20]. VAERS may be used to detect unexpected and rare patterns of AEFI, unlikely to arise in pivotal trials because of the limited number of patients involved [21]. Health-care professionals, vaccine manufacturers, and consumers (patients, parents, and caregivers) can submit reports of AEs to VAERS.

VigiBase^®^ is one of the largest and most comprehensive pharmacovigilance databases, maintained by the Uppsala Monitoring Centre in Sweden, and containing over 20 million of Individual Case Safety Reports from 110 countries over the five continents. VigiBase^®^ collects reports of AEs for authorized drugs, vaccines, and food supplements, submitted from healthcare professionals, pharmaceutical companies, and patients.

For each vaccine/drug, the characterization of the vaccine/drug role indicated by the primary reporter (i.e., the original source of the information) includes the following three categories: suspect, concomitant, and interacting. All spontaneous reports should have at least one suspect vaccine/drug, namely involved, presumably, in the occurrence of AEFI. If the reporter indicates a suspected interaction, all interacting vaccines/drugs are considered to be suspect vaccines/drugs.

In both databases, AEs are codified through the Medical Dictionary for Regulatory Activities (MedDRA) terminology, and described and organized in terms of Preferred Terms (PTs).

### 2.3. Data Extraction and Analysis

A multi-step approach was followed for data extraction in each database:

(1) Reports for individuals receiving any type of vaccine against the influenza virus categorized as suspect AEFI submitted to VAERS (from July 1990 to September 2020) and VigiBase^®^ (from inception to October 2020) were selected.
(2) Cases of myocarditis or pericarditis were extracted through specific PTs and lowest level terms, in line with previous studies on the influenza vaccination [4] and the potential immune-related basis of cardiotoxicity documented for ICIs by recent pharmacovigilance analyses [9,10]: myocarditis, pericarditis, immune-mediated myocarditis, and myopericarditis.
(3) Reports recording AEs of interest were finally assessed for co-reported drugs/vaccines of interest.

In VigiBase, retained reports (refer to point 2) with ICIs, namely PD-1 inhibitors (nivolumab, pembrolizumab, and cemiplimab), PD-L1 inhibitors (atezolizumab, avelumab, and durvalumab), and CTLA-4 inhibitors (ipilimumab and tremelimumab), were evaluated by extracting the following data: demographic features (age, gender, weight, year, and reporter country), reporter qualification, comorbidities, concomitant medications (including dose and route of administration when available), latency, degree of seriousness, outcome, and management of the AE. The same approach was applied to identify cases of interest reported for vaccines against the influenza virus, both as suspect alone or concomitant to ICI exposure.

In VAERS, for each report of interest with the influenza vaccines, the following data were extracted: demographic features (age, gender, year, and reporter country), type of vaccine against the influenza virus, medical history, concomitant drugs or administered vaccines, laboratory data, AEs codified as PTs, latency, AE degree of seriousness, outcome, and narratives, when available.

According to WHO criteria, a serious AE is any untoward medical occurrence that, no matter the dose, results as fatal, causing a life-threatening event, requiring hospitalization of the patient, causing serious/permanent disability, causing congenital abnormalities, or other clinically relevant conditions [22].

Co-reported medications and comorbidities known to cause myocarditis or pericarditis (i.e., potential confounders) were further characterized according to the lists proposed by Adler et al. [6], Caforio et al. [7], and Butany et al. [8].

### 2.4. Causality Assessment and Evaluation of Drug–Vaccine Interaction

The probability of interaction when the influenza vaccine and ICIs are co-reported was evaluated by applying the established WHO criteria for causality assessment [20], as well as the Drug Interaction Probability Scale (DIPS) [23].

The following items were accounted for in the AEFI causality assessment: temporal relationship, alternate explanations, proof of association, prior evidence, population-based evidence, and biological plausibility [20]. Cases without adequate information were classified as “unclassifiable”; cases with adequate information were categorized as: (1) “consistent with a causal relationship”, when the available evidence supported a causal relationship between the vaccine and the AEFI in the individual, but it did not rule out the possibility that the AEFI may have been caused by a factor other than the vaccine; (2) “inconsistent with a causal association”, when the available evidence did not support a causal relationship between vaccine administration and the reported AEFI in the individual; and (3) “indeterminate”, when the temporal relationship was consistent but the available evidence insufficient to support or rule out a causal relationship in the individual.

The DIPS is a 10-item tool specifically developed to assess the likelihood of drug–drug interactions [23]. ICIs were considered as the objective drugs (i.e., the one affected by the presence of another vaccine/drug), while the influenza vaccine was considered as the precipitant agent (i.e., the one causing a change on the object drug). To avoid overemphasis on the role of the vaccine, the answer to the second question was always “unknown” (knowledge on the mechanism of the interaction is hypothesized and literature data are very scarce/uncertain); in addition, questions 5, 6, and 10 were not assessable/applicable (dechallenge, rechallenge, and dose adjustments are unfeasible for vaccines considering the peculiarities of their administration), as previously performed [3]. The final summary score could reach 10 points. Higher total scores correspond to a higher likelihood of drug–vaccine interaction (i.e., >8 = highly probable; 5–8 = probable; 2–4 = possible; <2 = doubtful).

## 3. Results

### 3.1. Demographic and Clinical Data

Over the observed period, out of a total of 712,776 AEFI, 191 (0.03%) reports of MP mentioning the influenza vaccine as suspect were collected within the VAERS.

The clinical and demographic characteristics are provided in Table 1. The reports included 124 men (64.9%) and 62 women (in five cases gender was not reported), aged between 1 and 88 years (mean age 44.5 years). Pericarditis were reported in 117 cases (61.3%), followed by myocarditis (81 cases; 42.4%) and MP (7 cases; 3.7%). No cases of immune-mediated myocarditis were retrieved.

The median onset was seven days; in 34.0% of reports, the AEFI of interest occurred in the first 3 days after the administration of the influenza vaccine. Comorbidities potentially implicated in the occurrence of the AEFI of interest were retrieved in six cases (3.1%), with ulcerative colitis and systemic lupus erythematosus being the most represented. Other vaccines were concomitantly administered in 59 cases (30.9%), accounting for a mean number of 3.1 vaccines per patient.

In 141 cases (73.8%), the report was classified as serious, and 16 fatal cases (8.4%) were found. Recovery occurred in 31.4% of cases.

In the VigiBase^®^, 246,864 reports mentioning the influenza vaccine as a suspect agent were found, and myocarditis/pericarditis were reported in 399 cases (0.16%; Table 1).

The cases showed a mean age of 46.7 years, with a male preponderance (65.9%). Reports were largely submitted from US (45.9%) and Europe (45.1%). Pericarditis was reported in 225 cases (56.4%), followed by myocarditis (193 cases; 48.4%) and myopericarditis (29; 7.3%). No cases of immune-mediated myocarditis were found.

Median onset was five days, and in 29.8% of reports, AEs of interest occurred in the first three days after the administration of the influenza vaccine. Comorbidities potentially implicated in the occurrence of myocarditis/pericarditis were retrieved in two patients (0.5%) affected by ulcerative colitis. Other vaccines were concomitantly administered in 87 cases (21.8%), accounting for a mean number of 2.5 vaccines per patient.

In 303 cases (76.0%), the report was classified as serious, and 28 fatal cases (7.0%) were found. Recovering occurred in 41.1% of cases.

### 3.2. Co-Reported Medications and Detection of Cases with ICI Administration

Overall, concomitant medications were found in 49 (25.7%) and 57 (14.3%) cases with the influenza vaccine in VAERS and VigiBase^®^, respectively (Table 1). In 14 (7.3%) and 13 (3.3%) cases, five or more concomitant drugs were reported in VAERS and VigiBase^®^, respectively.

Concomitant medications potentially implicated in the occurrence of myocarditis/pericarditis were retrieved in 21 (11.0%) and 24 (6.0%) cases in VAERS and VigiBase^®^, respectively, with hydrochlorothiazide being the most frequent in both databases (in six and five cases, respectively).

Only six cases (one classified as serious, where Guillain-Barré syndrome was recorded) with the concomitant use of ICIs and the influenza vaccine were found in VAERS (Table S1), but no cases of myocarditis/pericarditis. In VigiBase^®^, two cases of myocarditis reporting the concomitant administration of ICIs and the influenza vaccine both mentioned as suspect agents were retrieved. A third case of myocarditis in which the influenza vaccine was classified as a concomitant agent was detected (Table 2).

The three cases were classified as serious (causing prolonged hospitalization), included two men and one woman, aged 67–77 years, and affected by lung or renal carcinoma. Nivolumab, pembrolizumab, and ipilimumab/nivolumab combination therapy were reported as the ICI regimen. In two cases ICI was withdrawn, while recovering occurred in only one case.

### 3.3. Causality Assessment and Evaluation of Drug–Vaccine Interaction

These three cases showed an unclassifiable causality assessment due to the lack of data concerning the latency and time window of increased risk (Table 2).

By applying the adapted DIPS algorithm, one report was categorized as possible (due to the lack of underlying diseases or co-reported agents known to cause or precipitate myocarditis, and to the detection of troponin increase) and two as doubtful (only the absence of underlying diseases or co-reported agents known to cause or precipitate myocarditis were recognized; Table S2).

## 4. Discussion

To the best of our knowledge, this is the first real-world study investigating the potential interactions between influenza vaccines and ICIs resulting in myocarditis or pericarditis in cancer patients, by assessing spontaneous reports submitted to the VAERS and VigiBase^®^. This global post-marketing safety study stems from recent conflicting real-world evidence surrounding the possible exacerbation of irAEs with influenza vaccines in patients treated with ICIs [14,15,24,25,26,27,28,29], thus joining in the wider debate on the bidirectional relationship between immunotherapy and the influenza vaccination, potentially affecting the clinical and humoral efficacy of the vaccine, performance of ICIs, and safety [14].

Four major findings emerged from our analysis: (a) myocarditis and pericarditis represented a very rare AEFI (with a non-negligible proportion of death, approximately 8–9%), based on the low reporting frequency retrieved in both SRSs (<0.1–0.2% of overall AEs), also considering the influenza vaccination coverage rates (estimated at about 25% per year in Europe [30,31]) and the relevant million doses distributed worldwide; (b) the reporting of myocarditis in patients concomitantly receiving influenza vaccines and ICIs is limited to only three out of a total of 1465 cases of myocarditis/pericarditis found with influenza vaccines or ICIs (considered as suspect agents) in the two databases; (c) these cases were unclassifiable for causality assessment with a doubtful probability of drug–vaccine interaction (according to the adapted DIPS algorithm), thus suggesting that the vaccine is not directly involved in the occurrence of myocarditis (and in one of the three cases, influenza vaccine was reported only as concomitant and not as suspect or interacting agent); and (d) no other cardiovascular AEs were found in patients concomitantly receiving influenza vaccination and ICIs, as well as no other irAEs were reported, except for a single case of Guillain-Barré syndrome, a condition already associated with both ICIs and the influenza vaccine [32,33,34].

Theoretically, a possible pharmacodynamic interaction between influenza vaccines and ICIs leading to the exacerbation of irAEs (including MP), could be supposed. The blockade of a PD-1/PD-L1 pathway together with the vaccination (particularly in conjunction with a strong vaccine adjuvant) could enhance one or more of the mechanisms associated with irAEs onset (infiltration of central memory T cells into the tissues, cross-presentation of shared antigens, and exacerbation of previously subclinical auto-immune syndromes) [24].

Our specific focus on the myo-pericardium stems from the peculiar clinical features of myocarditis in terms of severity and mortality compared with other irAEs, usually requiring immunotherapy discontinuation [9,10,11,12,13], as reported in two out of our three cases. Although, in all three cases, no underlying diseases or other agents known to cause or precipitate myocarditis were recorded, the lack of data concerning latency and solid literature studies allows for minimizing, if not completely excluding, the clinically relevant contribution of a potential drug–vaccine interaction in our cases.

Furthermore, the only study investigating the association between influenza vaccine and the development of myocarditis among patients on ICIs found that the administration of the vaccine was not associated with an increased risk of subsequent myocarditis [15]. Additionally, myocarditis cases in which the influenza vaccine was administered showed lower troponin levels at presentation and a lower risk of major adverse cardiac events at follow-up compared with non-vaccinate patients developing myocarditis with ICIs [15], thus suggesting a protective role for the influenza vaccine in this setting.

ICIs caused a paradigm shift in cancer treatment, and are currently used as first-line agents in the management of non-small cell lung cancer, melanoma, and renal carcinoma [35]. Considering their evolving role and expected increasing uptake, the assessment of cardiovascular safety is of paramount importance. Likewise, the safety of influenza vaccines represents an important issue, given the high morbidity and mortality rates caused by influenza in cancer patients [36]. Notably, the development of influenza infection may also, albeit rarely, be associated itself with an increased risk of myocarditis and major adverse cardiovascular events [37,38]. Therefore, the suggested protective role of the influenza vaccine on cardiovascular outcome [15] cannot be overlooked.

Collectively, our findings provide a reassuring message in terms of cardiovascular safety for cancer patients treated with ICIs and requiring the influenza vaccination. Of note, less than 20% of patients affected by malignancies received the influenza vaccine, with a gradual decline in the last decade [39]. We support and promote the achievement of an optimal vaccination coverage rate in cancer patients for several reasons, including the following: (a) direct association with a lower mortality and infection-related outcomes in immunosuppressed adults [1]; (b) a better overall survival recently reported in patients treated with ICIs receiving influenza vaccination [40]; and (c) the current COVID-19 pandemic, to reduce the strain on the healthcare system while protecting vulnerable subjects from the dramatic impact of a possible co-infection [41]. Cancer patients receiving immunotherapy are at high risk of severe events as a result of COVID-19 systemic involvement, including pneumonitis and myocarditis [42], and a recent systematic review found no significant increase in the risk of infection or in the illness severity or lethality of COVID-19 in subjects receiving the influenza vaccine, with some studies reporting a significantly inverse association [43]. Therefore, the implementation of measures aimed at raising influenza vaccination coverage in frail patients is strongly recommended.

We acknowledge the limitations of our study, mainly inherent to the nature of SRSs data. VAERS and VigiBase^®^ are subject to reporting bias, including under- and over-reporting of adverse events, although there are not clues for major distortions, considering that serious events such as MP are less prone to under-reporting, and no specific warnings were posted by regulatory agencies, thus minimizing the existence and impact of a stimulated reporting [3,18]. We recognize the potential occurrence of subclinical myocarditis, which may be under-diagnosed/under-detected, and is thus likely to be under-reported. Furthermore, the quality and completeness of the reports collected in both databases are variable, and many records lack valid medical diagnoses, thus making the assessment of causality challenging. Additionally, DIPS was not specifically developed to assess drug–vaccine interactions, although we implemented an adapted version in order to the better focus on the possible precipitating role of the influenza vaccine.

Notwithstanding these limitations, pharmacovigilance assessment represents an invaluable opportunity to monitor vaccine safety and identify novel rare signals, potentially arising from drug–vaccine interactions, both from a local and international perspective. Furthermore, our findings are consistent between the two databases, thus supporting the lack of evidence of a clinically relevant drug–vaccine interaction. The identification of myocarditis with ICIs, in line with previous findings [9,10,11], further corroborates the ability of our post-marketing approach to identify actual true-positive associations.

In conclusion, the paucity of cases coupled with a lack of certainty in terms of causality assessment and doubtful probability make the risk of myocarditis and pericarditis by interaction between influenza vaccines and ICIs in cancer patients negligible from both clinical and epidemiological standpoints. Our findings support the cardiovascular safety of influenza vaccines in subjects treated with immunotherapy, thereby emphasizing the importance of a flu vaccination in this population, especially in the current COVID-19 era.

## Figures and Tables

**Table 1:** Demographic and clinical feature of cases of myocarditis/pericarditis reported with influenza vaccine as suspect in Vaccine Adverse Event Reporting System (VAERS) and the World Health Organization’s (WHO) global Individual Case Safety Report database (VigiBase^®^).

Demographic Features	VAERS	VigiBase
Overall number of cases	191	399
Proportion of cases (based on overall number of reports with the flu vaccine)	0.03% (712,776)	0.16% (246,864)
Age (mean)	44.5 ± 22.9	46.7 ± 22.3
*Sex*		
Female	62 (32.5%)	131 (32.8%)
Male	124 (64.9%)	263 (65.9%)
Not specified	5 (2.6%)	5 (1.3%)
*Reporter country*		
US	109 (57.1%)	183 (45.9%)
Non-US	60 (31.4%)	-
Europe	-	180 (45.1%)
Asia	-	10 (2.5%)
Oceania	-	19 (4.8%)
America (except US)	-	7 (1.7%)
Not specified	22 (11.5%)	-
*Reported symptoms (preferred terms)* *		
Myocarditis	81 (42.4%)	193 (48.4%)
Pericarditis	117 (61.3%)	225 (56.4%)
Myopericarditis	7 (3.7%)	29 (7.3%)
Immune-mediated myocarditis	0 (0.0%)	0 (0.0%)
*Co-medications*		
Overall number of cases	49 (25.7%)	57 (14.3%)
≥5 concomitant drugs	14 (7.3%)	13 (3.3%)
1–4 concomitant drugs	35 (18.4%)	44 (11.0%)
No concomitant drugs	142 (74.3%)	342 (85.7%)
*Co-medications potentially implicated in the occurrence of myocarditis/pericarditis* **		
Number of cases	21 (11.0%) ^§^	24 (6.0%) ^#^
Immune checkpoint inhibitors (ICIs)	None	2
Hydrochlorothiazide	6	5
Indomethacin	2	2
Glipizide	2	-
Clonazepam	2	-
Alprazolam	2	-
Furosemide	1	1
Bendroflumethiazide	1	2
Mesalazine	1	2
Colchicine	1	1
Doxycycline	1	2
Cotrimoxazole	1	1
Lorazepam	1	-
Amoxicillin	1	1
Isosorbide dinitrate	1	2
Tetracycline	-	2
Spironolactone	-	1
Cefuroxime	-	1
Heparin	-	1
Bromazepam	-	1
*Comorbidities potentially implicated in occurrence of myocarditis/pericarditis* **		
Number of cases	6 (3.1%)	2 (0.5%)
Ulcerative colitis	2	2
Systemic lupus erythematosus	2	-
Juvenile rheumatoid arthritis	1	-
Insulin-dependent diabetes mellitus	1	-
*Onset* (days; median)	7 (1.5–13)	5 (1–12)
≤3 days	65 (34.0%)	119 (29.8%)
4–7 days	19 (10.0%)	50 (12.5%)
8–14 days	35 (18.4%)	61 (15.3%)
≥15 days	36 (18.8%)	56 (14.0%)
Not specified	36 (18.8%)	113 (28.4%)
*Seriousness*		
Serious	141 (73.8%)	303 (76.0%)
Non-serious	50 (26.2%)	48 (12.0%)
Not specified	-	48 (12.0%)
*Seriousness criteria* *		
Congenital anomaly	0 (0.0%)	0 (0.0%)
Death	16 (8.4%)	28 (7.0%)
Hospitalization	125 (65.5%)	222 (55.6%)
Life-threatening	30 (15.7%)	46 (11.5%)
Permanent disability	12 (6.3%)	12 (3.0%)
Other outcomes	30 (15.7%)	60 (15.0%)
Median time of hospitalization (days)	3 (2–4)	NA
*Recovering*		
Recovered	60 (31.4%)	164 (41.1%)
Not recovered	64 (33.5%)	61 (15.3%)
Not specified	67 (35.1%)	174 (43.6%)
*Concomitant other vaccines*		
Overall number of cases	59 (30.9%)	87 (21.8%)
Mean number of vaccines per patient	3.1 ± 1.4	2.5 ± 1.7

* One case may exhibit more than one seriousness criteria. ** According to 2015 the European Society of Cardiology (ESC) guidelines for the diagnosis and management of pericardial diseases [6], Caforio et al. [7] and Butany et al. [8]. ^§^ In one case, the concomitant use of glipizide and indomethacin, and colchicine and indomethacin. ^#^ In one case, concomitant use of amoxicillin and tetracycline, hydrochlorothiazide and cotrimoxazole, and bromazepam and heparin. Flu vaccine types (VAERS): FLU 3 (trivalent injected): 69 patients (36.1%); FLUX SEASONAL (influenza virus vaccine, no brand name): 62 (32.5%); FLU 4 (quadrivalent injected): 20 (10.5%); FLUN 3 (trivalent intranasal spray): 19 (9.9%); FLUN 4 (quadrivalent intranasal spray): 7 (3.7%); FLU H1N1 (monovalent injected): 5 (2.6%); FLUA 3 (trivalent adjuvant injected): 3 (1.6%); FLUN H1N1 (monovalent intranasal spray): 2 (1.1%); FLUX H1N1 (monovalent unknown manufacturer): 2 (1.1%); FLUC 4 (quadrivalent cell-culture-derived injected): 2 (1.1%); FLUC 3 (trivalent cell-culture-derived injected): 1 (0.5%). One patient received both FLUX SEASONAL and FLUX (H1N1).

**Table 2:** Case-by-case assessment of reports concerning myocarditis or pericarditis in which the influenza vaccine and immune checkpoint inhibitors (ICIs) were concomitantly used.

Case ID	Drugs/Role	Dose	Year	Age/Sex	Reporter Country	Reporter Qualification	Reactions	Seriousness	Outcome	Concomitant Medications	Comorbidities	Management	Causality Assessment	Adapted DIPS Score
#1	Nivolumab (suspect) Influenza vaccine(suspect)	3 mg/kg every 2 weeks IV-	2018	70/F	Japan	Physician	Myocarditis	Serious (life-threatening; prolonged hospitalization; other outcomes)	Recovered	Ursodeoxycholic acid 200 mg/day Bezafibrate 200 mg/day Calcitriol 0.5 mcg/day	NSCLC Liver disorder Hyperlipidemia Osteoporosis	Nivolumab withdrawn	Unclassifiable (data on latency and time window of increased risk are lacking) Synergistic effect between influenza vaccine and ICIs cannot be excluded	1 Doubtful
#2	Pembrolizumab (suspect) Influenza vaccine (suspect)	NA IV 0.5 mL IM	2018	67/M	US	Pharmacist	Myocarditis, stress, cardiomyopathy, weight decreased, headache, cardiac failure, congestive, cerebral infarction, confusional state, and dyspnoea	Serious (life-threatening; prolonged hospitalization)	NA	NA	Lung neoplasm malignant	NA	Unclassifiable (data on latency and time window of increased risk are lacking) Synergistic effect between influenza vaccine and ICIs cannot be excluded	1 Doubtful
#3	Ipilimumab (suspect) Nivolumab (suspect) Influenza vaccine (concomitant)	80 mg every 3 weeks IV 240 mg every 3 weeks IV-	2019	77/M	Canada	Physician	Myocarditis, pulmonary hypertension, dyspnoea chest discomfort asthenia, troponin increased c-reactive protein, increased diastolic dysfunction, oedema, peripheral urticaria, and pruritus	Serious (prolonged hospitalization; other outcomes)	NA	NA	Metastatic renal cell carcinoma	Ipilimumab and nivolumab withdrawn	Unclassifiable (data on latency and time window of increased risk are lacking) Synergistic effect between influenza vaccine and ICIs cannot be excluded	2 Possible

US—United States of America; NSCLC—non-small cell lung cancer; NA—not available; IV—intravenous; IM—intramuscular; DIPS—drug-interaction probability scale.

## Data Availability

Data supporting the findings of this study were derived from the following resource available in the public domains: https://wonder.cdc.gov/vaers.html and http://www.vigiaccess.org/.

## Supplementary Materials

The following are available online at https://www.mdpi.com/2076-393X/9/1/19/s1. Table S1: Case-by-case assessment of reports recorded in VAERS in which influenza vaccine and immune checkpoint inhibitors (ICIs) were concomitantly used. Table S2: Application of the adapted version of Drug Interaction Probability Scale in cases of myocarditis/pericarditis in which immune checkpoint inhibitors (ICIs) and influenza vaccine were concomitantly administered.
