# Supplementary material for: Influenza Vaccination and Myo-Pericarditis in Patients Receiving Immune Checkpoint Inhibitors: Investigating the Likelihood of Interaction through the Vaccine Adverse Event Reporting System and VigiBase
Source: Vaccines (Basel). 2021 Jan 4;9(1):19. doi: 10.3390/vaccines9010019 (PMC7823897; doi:10.3390/vaccines9010019)
Supplement: Supplementary file 1 [file vaccines-09-00019-s001.pdf]

Influenza vaccination and myo-pericarditis in patients receiving immune checkpoint inhibitors: investigating the likelihood of interaction through the Vaccine Adverse Event Reporting System and VigiBase

Milo Gatti, Emanuel Raschi, Ugo Moretti, Andrea Ardizzoni, Elisabetta Poluzzi, Igor Diemberger

SUPPLEMENTARY MATERIALS

Supplementary Table 1 – Case-by-case assessment of reports recorded in VAERS in which influenza vaccine and immune checkpoint inhibitors (ICIs) were concomitantly used.

Supplementary Table 2 – Application of the adapted version of Drug Interaction Probability Scale in cases of myocarditis/pericarditis in which immune checkpoint inhibitors (ICIs) and influenza vaccine were concomitantly administered.

Supplementary Table 1 – Case-by-case assessment of reports recorded in VAERS in which influenza vaccine and immune checkpoint inhibitors (ICIs) were concomitantly used.

Case ID	Drugs	Year	Age/Sex	Reporter country	Reporter qualification	Time to onset (days)	Reactions	Seriousness	Outcome	Concomitant medications	Comorbidities	Management
#1	Influenza vaccine<br><br>Pembrolizumab	2018	NA/M	NA	Physician	NA	Allergy to vaccine	Not serious	NA	Oxycodone<br>Ondansetron<br>Prochlorperazine	Mesothelioma	NA
#2	Influenza vaccine<br><br>Pembrolizumab	2018	63/F	US	Physician	1	Fall<br>Head injury<br>Loss of consciousness<br>Lower limb fracture<br>Nausea	Not serious	Recovered	Prednisone<br>Bevacizumab<br>Atorvastatin<br>Losartan<br>Pregabalin<br>Bisacodyl<br>Selenium<br>Vitamin B2	NA	NA
#3	Influenza vaccine<br><br>Nivolumab	2019	71/M	US	Physician	4 hours	Chills<br>Pyrexia<br>Vomiting	Not serious	Recovered	Gabapentin<br>Triamterene<br>Hydrochlorothiazide<br>Levothyroxine<br>Carbamazepine<br>Phenytoin<br>Budesonide/Formoterol	Seizure<br>COPD<br>Renal cell carcinoma metastazied to lung<br><br>Previous reaction to influenza vaccine four years ago	Anti-emetics<br>Fluids
#4	Influenza vaccine<br><br>Nivolumab	2019	82/M	US	Physician	1	Feeling cold<br>Muscular weakness<br>Nausea<br>Tremor<br>Vomiting	Not serious	Recovered	Levothyroxine<br>Prednisone<br>Doxazosin<br>Pneumococcal vaccine	Hypothyroidism<br>Cancer not specified	NA
#5	Influenza vaccine<br><br>Nivolumab	2018	67/M	NA	Physician	NA	Alanine aminotransferase increased<br>Aspartate aminotransferase increased<br>Blood alkaline phosphatase increased<br>Blood bilirubin normal<br>Blood calcium normal<br>Blood chloride increased<br>Blood creatinine normal	Not serious	Not recovered	Pazopanib	Hypertension<br>Clear cell renal cell carcinoma<br>Metastases to lung<br>Alcohol use<br>Metastases to bone<br>Prostate cancer<br>Malignant melanoma	NA

							Blood potassium normal<br>Blood sodium normal<br>Blood urea increased<br>Carbon dioxide normal<br>Cough<br>Dyspnoea<br>Haematocrit decreased<br>Haemoglobin decreased<br>Mean cell volume normal<br>Neurological examination normal<br>Platelet count normal<br>Psychiatric evaluation normal<br>Radiation pneumonitis<br>Rales<br>Sleep disorder<br>Toxicity to various agents<br>Weight decreased<br>White blood cell count normal					
#6	Influenza vaccine<br><br>Nivolumab	2019	66/M	NA	Physician	21	Anti-neuronal antibody<br>Antibody test negative<br>Areflexia<br>Asthenia<br>Autonomic nervous system imbalance<br>Blood pressure fluctuation<br>Bradycardia<br>Computerised tomogram abdomen abnormal<br>Computerised tomogram pelvis abnormal<br>CSF lymphocyte count normal<br>CSF monocyte count increased<br>CSF protein increased<br>CSF red blood cell count positive	Serious (death)	Not recovered	-	Metastatic melanoma	Intravenous immunoglobulin<br>Prednisone

							CSF white blood cell count increased<br>Cytology normal<br>Death<br>Decreased vibratory sense<br>Demyelinating polyneuropathy<br>Electromyogram abnormal<br>Endotracheal intubation<br>Gait inability<br>Guillain-barre syndrome<br>Heart rate abnormal<br>Hepatic mass<br>Hypotension<br>Immunoglobulin therapy<br>Mechanical ventilation<br>Metastases to central nervous system<br>Muscular weakness<br>Nerve conduction studies abnormal<br>Nuclear magnetic resonance imaging brain abnormal<br>Paraesthesia<br>Plasmapheresis<br>Respiratory distress<br>Sensory loss<br>Tachycardia<br>Walking aid user<br>Withdrawal of life support					

CSF: cerebrospinal fluid; COPD: chronic obstructive pulmonary disease; NA: not available

Supplementary Table 2 – Application of the adapted version of Drug Interaction Probability Scale in cases of myocarditis/pericarditis in which immune checkpoint inhibitors (ICIs) and influenza vaccine were concomitantly administered.

**Items**	**Case #1**<br>(Influenza vaccine-Nivolumab)	**Case #2**<br>(Influenza vaccine-Pembrolizumab)	**Case#3**<br>(Influenza vaccine-Nivolumab/Ipilimumab)
**#1**	NA (0 pts)<br>A retrospective case-control study [15] reported cases of myocarditis in patients treated with ICIs exposed to influenza vaccine, although less severe compared to cases of myocarditis in non-vaccinate patients	NA (0 pts)<br>A retrospective case-control study [15] reported cases of myocarditis in patients treated with ICIs exposed to influenza vaccine, although less severe compared to cases of myocarditis in non-vaccinate patients	NA (0 pts)<br>A retrospective case-control study [15] reported cases of myocarditis in patients treated with ICIs exposed to influenza vaccine, although less severe compared to cases of myocarditis in non-vaccinate patients
**#2**	NA (0 pts)<br>A possible PD mechanism of the interaction could be theoretically hypothesized although literature data are lacking	NA (0 pts)<br>A possible PD mechanism of the interaction could be theoretically hypothesized although literature data are lacking	NA (0 pts)<br>A possible PD mechanism of the interaction could be theoretically hypothesized although literature data are lacking
**#3**	NA (0 pts)<br>A possible PD mechanism of the interaction could be theoretically hypothesized although literature data are lacking	NA (0 pts)<br>A possible PD mechanism of the interaction could be theoretically hypothesized although literature data are lacking	NA (0 pts)<br>A possible PD mechanism of the interaction could be theoretically hypothesized although literature data are lacking
**#4**	NA (0 pts)<br>Onset time was not available for this report	NA (0 pts)<br>Onset time was not available for this report	NA (0 pts)<br>Onset time was not available for this report
**#5**	NA (0 pts)<br>Dechallenge is unfeasible for vaccines considering the peculiarities of their administration	NA (0 pts)<br>Dechallenge is unfeasible for vaccines considering the peculiarities of their administration	NA (0 pts)<br>Dechallenge is unfeasible for vaccines considering the peculiarities of their administration
**#6**	NA (0 pts)<br>Rechallenge is unfeasible for vaccines considering the peculiarities of their administration	NA (0 pts)<br>Rechallenge is unfeasible for vaccines considering the peculiarities of their administration	NA (0 pts)<br>Rechallenge is unfeasible for vaccines considering the peculiarities of their administration
**#7**	No (+1 pts)<br>No underlying diseases or concomitant drugs known to cause or precipitate myocarditis were reported	No (+1 pts)<br>No underlying diseases or concomitant drugs known to cause or precipitate myocarditis were reported	No (+1 pts)<br>No underlying diseases or concomitant drugs known to cause or precipitate myocarditis were reported
**#8**	NA (0 pts)<br>PD interactions usually do not entail changes in the concentrations of the object drug, and nivolumab concentrations were not monitored	NA (0 pts)<br>PD interactions usually do not entail changes in the concentrations of the object drug, and pembrolizumab concentrations were not monitored	NA (0 pts)<br>PD interactions usually do not entail changes in the concentrations of the object drug, and ipilimumab/nivolumab concentrations were not monitored
**#9**	NA (0 pts)<br>No other evidence of the interaction was reported	NA (0 pts)<br>No other evidence of the interaction was reported	Yes (+1 pts)<br>Increase in troponin concentration was reported
**#10**	NA (0 pts)<br>Dose adjustments is unfeasible for vaccines considering the peculiarities of their administration	NA (0 pts)<br>Dose adjustments is unfeasible for vaccines considering the peculiarities of their administration	NA (0 pts)<br>Dose adjustments is unfeasible for vaccines considering the peculiarities of their administration
**Total score**	**1 (Doubtful)**	**1 (Doubtful)**	**2 (Possible)**

ICIs were considered as the objective drugs (i.e., the one affected by the presence of another drug), while influenza vaccine was considered as the precipitant drug (i.e., the one causing a change on the object drug).

PD: pharmacodynamic; NA: unknown or non-applicable.
